# Electro-Deformation of Fused Cells in a Microfluidic Array Device

**DOI:** 10.3390/mi7110204

**Published:** 2016-11-09

**Authors:** Yan Liu, Xiaoling Zhang, Mengdi Chen, Danfen Yin, Zhong Yang, Xi Chen, Zhenyu Wang, Jie Xu, Yuanyi Li, Jun Qiu, Ning Hu, Jun Yang

**Affiliations:** 1 Key Laboratory of Biorheological Science and Technology, Chongqing University, Ministry of Education, and Key Laboratory of Vision Loss, Regeneration and Restoration, Chongqing, Bioengineering College, Chongqing University, Chongqing 400030, China; 20141902049@cqu.edu.cn (Y.L.); zhangxiaoling@cqu.edu.cn (X.Z.); 20161902047@cqu.edu.cn (M.C.); 20161902048@cqu.edu.cn (D.Y.); 20121913019t@cqu.edu.cn (X.C.); 2 Department of Laboratory Medicine, Southwest Hospital, Third Military Medical University, Chongqing 400038, China; zyang@tmmu.edu.cn; 3 College of Biomedical Engineering, Chongqing Medical University, Chongqing 400016, China; wangzhenyu20090306@gmail.com; 4 Chongqing Jinshan Science & Technology (Group) Co., Ltd., Chongqing 401120, China; kyxuj@jinshangroup.com (J.X.); liyy@jinshangroup.com (Y.L.); 5 Department of Information, Southwest Hospital, Third Military Medical University, Chongqing 400038, China; qiujun1982@gmail.com

**Keywords:** electro-deformation, microfluidic, fused cell, mechanical properties

## Abstract

We present a new method of analyzing the deformability of fused cells in a microfluidic array device. Electrical stresses—generated by applying voltages (4–20 V) across discrete co-planar microelectrodes along the side walls of a microfluidic channel—have been used to electro-deform fused and unfused stem cells. Under an electro-deformation force induced by applying an alternating current (AC) signal, we observed significant electro-deformation phenomena. The experimental results show that the fused stem cells were stiffer than the unfused stem cells at a relatively low voltage (<16 V). However, at a relatively high voltage, the fused stem cells were more easily deformed than were the unfused stem cells. In addition, the electro-deformation process is modeled based on the Maxwell stress tensor and structural mechanics of cells. The theoretical results show that a positive correlation is found between the deformation of the cell and the applied voltage, which is consistent with the experimental results. Combined with a numerical analysis and experimental study, the results showed that the significant difference of the deformation ratio of the fused and unfused cells is not due to their size difference. This demonstrates that some other properties of cell membranes (such as the membrane structure) were also changed in the electrofusion process, in addition to the size modification of that process.

## 1. Introduction

Cell fusion is a method to asexually fuse two or more cells and produce a mono- or multi-nucleated fused cell in vitro [1]. The fused cell has new genetic or biological properties, as it integrates genetic material (i.e., genome and extranuclear genes) from both parent cells. Present research on fused cells has focused on the reprogramming of somatic cells [2,3], genetic analysis [4], developing antibodies [5], cloning mammals, and cancer immunotherapy [6]. In the cell fusion process, the mechanical properties of fused cells change during the cell fusion and bilayer reconstitution process, due to the mobility of the lipid. In addition, the existence of a membrane protein would also affect the mobility of the lipid to change the mechanical properties of the fused cell membrane [7,8].

As the mechanical properties can reveal some important information regarding the fusion process, such as the membrane protein structure, and potential applications in cell separation or tumor cell detection based on the mechanical properties, several well-known tools have been used to measure the mechanical properties of cells, including optical tweezers [9,10,11], micropipette aspiration (MPA) [12], atomic force microscopy (AFM) [13], and electro-deformation (ED) [14,15,16,17,18]. Among these methods, ED is more amenable to lab-on-a-chip implementation and does not require complicated equipment. When biological cells are in a nonuniform electric alternating current (AC) field, Maxwell–Wagner polarization occurs, resulting in dielectrophoresis (DEP), electrorotation (ER), and electro-deformation. DEP can be used to trap cells in a non-uniform electric field. With an increase in the strength of the electric field, cells can be stretched, which is called electro-deformation [19].

In the research described above, cell deformation has been demonstrated to be a potent method of illuminating cell denaturation. Considering that fusion manipulation induces membrane reconstruction, membrane protein structure modifications, and intracellular substance integration, great changes in mechanical properties occur during this process. Detection of the mechanical properties of fused cells has the potential to reveal some important information and afford a potential unmarked fused cell separation technique. However, the mechanical properties of the fused cells have not been investigated. To detect the differences between the mechanical properties of fused stem cells and unfused stem cells, a discrete co-planar microelectrode device was designed to generate non-uniform electric fields. The cells on the chip were trapped on the microelectrode under DEP and then deformed under a sufficient electric field. The whole process of cell deformation was recorded by a charge-coupled device (CCD) camera connected to a microscope. In addition, numerical simulations were performed to model cell electro-deformation based on the Maxwell stress tensor and structural mechanics of the cells.

## 2. Materials and Methods

### 2.1. Cells and Media

Cells were maintained in a standard cell culture incubator (5% CO_2_, 95% humidity, 37 °C). Unfused stem cells were from a mouse embryonic stem cell (mESC) line (MESPU35) that was cultured with irradiated embryonic fibroblasts as the feeder. These cells were derived from 12.5 day ICR mouse embryos and c-ray irradiated to arrest cell division at the third passage of culture to maintain mESCs in the undifferentiated state. They were cultured in high-glucose dulbecco's modified eagle medium (H-DMEM, Hyclone, Logan, UT, USA) supplemented with 10% fetal bovine serum (FBS, Hyclone), 2 mmol/L glutamine, 100 μg/mL penicillin–streptomycin, 1000 IU/mL leukemia inhibitory factor, 0.1 mmol/L β-mercaptoethanol, and 0.1 mmol/L nonessential amino acid. Fused stem cells [20] were cultured in mESC culture media. When collecting the cells, they were trypsinized using 0.25/0.02% trypsin/Ethylenediaminetetraacetic acid (EDTA) for approximately 1 min, arrested by H-DMEM containing 10% FBS, centrifuged at 1000 rpm for 5 min, and washed in 0.01 mol/L PBS (pH 7.2). The medium used for electrodeformation experiments was a medium buffer with low electrical conductivity (~0.001 S·m^−1^). Before the experiments, the cells were washed three times and resuspended in the medium buffer at low densities (1 × 10^6^/mL).

### 2.2. Fabrication of the Microfluidic Device, Operation, and Data Analysis

A discrete co-planer vertical sidewall microelectrode device was used to produce the electro-deformation force. This device was designed and fabricated on a silicon-on-insulator (SOI) wafer [21]. The device contained a serpentine-shaped microchannel with 22,500 pairs of vertical sidewall microelectrodes patterned on two opposite vertical sidewalls of the microchannel, and could produce a non-uniform electric field in the microchannel.

**Figure 1:**
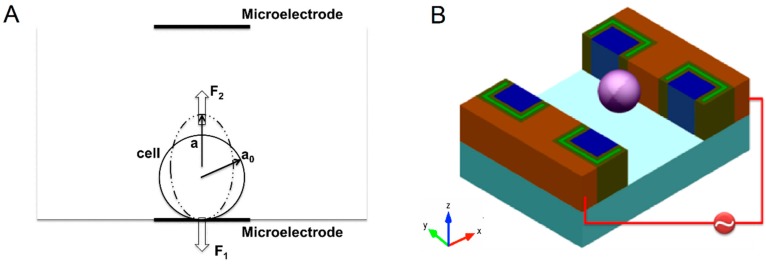
Schematic of electro-deformation (**A**) 2D and (**B**) 3D. The microelectrodes (red) on each sidewall are separated by coplanar SiO_2_ (dark green)–Polysilicon (light green)–SiO_2_ (dark green)/silicon (blue) insulators.

The operation procedures are described briefly as follows. The microchip was connected to an electrical signal generator. A sinusoidal electric potential of different amplitudes was applied to capture and deform cells. The microchannel was first rinsed and washed with fresh medium buffer. After loading, cells were randomly placed inside the microchannel. A small AC signal (1 V_p-p_, 1 MHz) was applied to attract cells to the electrodes due to DEP. The applied voltage was then increased 4 V per step from 4 V to 20 V and kept steady at each step for 60 s, and the cell behavior was observed and recorded with a CCD camera (Motic 3000, Motic, Xiamen, China) during the experiments. To measure the deformation ratio of each cell relative to the voltage applied, measurements of the cell dimensions were carried out manually using the ellipse-fitting and measurement tools of the Graphic Image Manipulation Program (GIMP v.2, The GIMP Development Team, International). The deformation ratio was defined as the ratio between the elongation of the cell parallel to the electric field after ED and the original radius of the cell before ED, which was calculated as

(1) $$\gamma =\frac{a}{a_{0}},$$

where *a*_0_ is the original radius of the cell and *a* is the length of the ellipse major axis after deformation, as shown in Figure 1.

### 2.3. Numerical Simulation of ED Process

Modelling of the ED process was conducted in COMSOL Multiphysics 4.3b (COMSOL, Inc., Palo Alto, CA, USA) using the *Electric Currents*, *Frequency domain*, and *Solid*, *Stationary* application modes. The cell was modeled as a sphere with radius *a*_0_. First, the distribution of the electric field was calculated in the microchannel, including the cell. Additionally, the ED forces exerted on the cell were computed by integrating the time-averaged Maxwell stress tensor over the cell surface [18]. Second, the cell was modeled as an incompressible linear elastic solid, which was described by a Neo-Hookean model [22]. *Solid* application mode was used to mimic the cell. A value of Young’s modulus and Poisson’s ratio of the cell was assumed, and the calculated ED forces were used as a load to calculate the cell deformation. Finally, the experiment results were used to fit the calculated deformations. The quantitative information used in the simulations is provided in Table 1.

The electric potential inside and outside the cell was obtained by solving the equation in the frequency domain [16]

(2) $$-\nabla \cdot \left(\left(\sigma_{\mathrm{out}}+j{\omega \varepsilon }_{0}\varepsilon_{\mathrm{out}}\right)\nabla \phi_{\mathrm{out}}\right)=0,$$

(3) $$-\nabla \cdot \left(\left(\sigma_{\mathrm{in}}+j{\omega \varepsilon }_{0}\varepsilon_{\mathrm{in}}\right)\nabla \phi_{\mathrm{in}}\right)=0,$$

where $\varepsilon_{0}$ is the permittivity of the vacuum; $\varepsilon_{\mathrm{out}}$ and $\sigma_{\mathrm{out}}$ are, respectively, the relative permittivity and the conductivity of the medium; and $\varepsilon_{\mathrm{in}}$ and $\sigma_{\mathrm{in}}$ are, respectively, the relative permittivity and the conductivity of the cytoplasm. ω is the angular frequency of the AC electric field. $j=\sqrt{-1}$ is the imaginary unit. The voltage for ED ($\Phi_{0}$) was applied via embedded discrete electrodes. Thus, the electrical boundary condition on the electrodes was assumed to be

(4) $$\Phi =\Phi_{0}\;\mathrm{or}\;0$$

The remaining walls of the microchannel were electrically insulated. The cell settled down the microelectrode (the highest electric field region) with forces *F*_1_ and *F*_2_ on two hemi-ellipsoids. These distributed forces on the cell led to elongation of the cell at an equilibrium location. The forces due to the electric field are calculated from the integration of the time-averaged Maxwell stress tensor over the cell’s surface,

(5) $$T=\frac{\varepsilon_{0}\varepsilon_{\mathrm{out}}}{4}\left(EE^{*}+E^{*}E-{\left|E\right|}^{2}I\right),$$

which is responsible for the steady deformation of the cell. **T** is the time-averaged Maxwell stress tensor, **E** is the applied external electric field, E* is the complex conjugate of **E**. **I** is the unit tensor. For trapped cells in the non-uniform electric field, we assumed that $\pm \left|F_{1}-F_{2}\right|/2$ were the forces acting on each half-sphere for electro-deformation, which stretched the cell approximately uniaxially. The total electroformation force is defined as

(6) $$F=\left|F_{1}-F_{2}\right|.$$

At the cell membrane, a *Distributed Impedance* boundary condition was set to introduce the influence of the cell membrane:

(7) $$n\cdot \left(J_{1}-J_{2}\right)=\frac{\left(\sigma_{m}+j{\omega \varepsilon }_{0}\varepsilon_{m}\right)}{d_{m}}\left(V-V_{\mathrm{ref}}\right).$$

Some constraints were set to prevent the cell from shifting in space; that is, the pole at the microelectrode cannot move at all, the lines at the *x–y* plane cannot move along the *z* axis, and the lines at the *x–z* plane cannot move along the *y* axis.

## 3. Results and Discussion

### 3.1. Simulation Results

**Figure 2:**
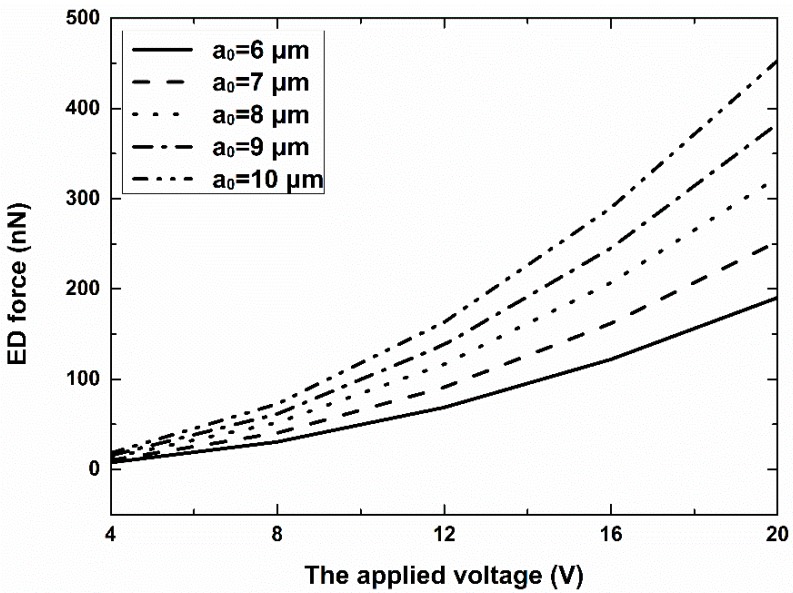
Electro-deformation (ED) forces as a function of the applied voltage and radius of the cell. The applied frequency was 1 MHz.

**Figure 3:**
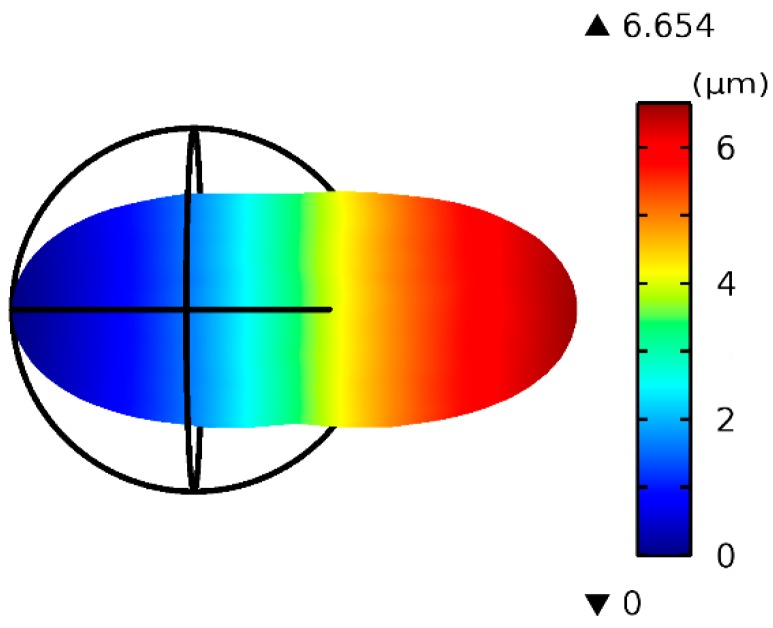
The deformation of a cell with $a_{0}$ = 6 μm under 16 V AC. The color represents the displacement along the *x* axis.

As the electric properties of cells are mostly unknown, we used common values. As shown in Figure 2, as the applied voltage increased from 4 V to 20 V, the electrodeformation force changed from 7.6 nN to 190.4 nN for a cell with $a_{0}$ = 6 μm. Larger cells experience a larger electro-deformation force, but larger cells also require a large force to deform. Figure 3 depicts an example of the deformation of a cell with radius $a_{0}$ = 6 μm under 16 V of AC voltage when the Young’s modulus of the cell is 600 Pa. The color represents the displacement along the *x* axis. The deformation ratio is ~1.555.

### 3.2. Cell Elongation

Firstly, a small AC signal (~1 V_p-p_, 1 MHz) was applied on the microelectrode array to produce a non-uniform electric field. Considering that the relative permittivity of the cell sample was higher than the surrounding medium, the cells would move to the electrode under positive-DEP force induced by the non-uniform electric field. To avoid cell alignment phenomenon effects on cell electrodeformation detection, cells were loaded at a low density. After the cells were stably located at the desired place (attached to the microelectrode), AC signals (4–20 V) with different amplitudes were chosen to electrically deform the cells.

In addition to the amplitude, the frequency of the AC signals was also a very important parameter for cell electrodeformation. In DC or low frequency fields, most of the applied voltage drops across the cell membrane, so cell lysis is easy to occur. Whereas at very high frequencies, small electrodynamic forces are generated because the cell membrane becomes electrically transparent [16]. In our experiments, we chose a frequency of 1 MHz, which generated high electrodynamic forces and also reduced the electrolysis effect.

**Figure 4:**
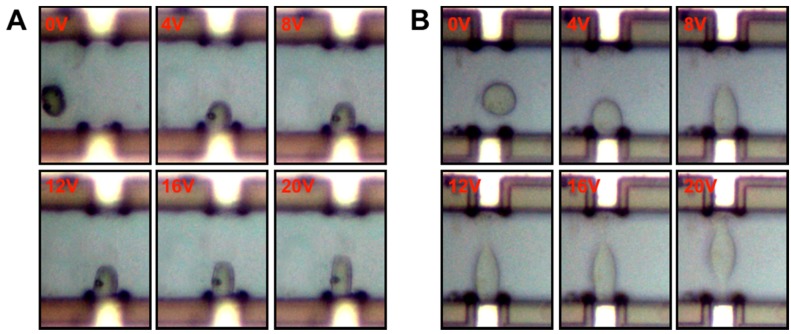
Deformation of (**A**) the unfused stem cell and (**B**) the fused stem cell as a function of the electric field strength.

When subjected to electric fields, both the fused and unfused stem cells showed deformation parallel to the applied electric field lines, as shown in Figure 4. With the increase in applied voltage, the deformation degree also increased. When the applied voltage was significantly high, some cells could be very deformed and cross the middle of the microchannel, or even move to the opposite microelectrode.

### 3.3. Comparison of the Fused and Unfused Stem Cells

**Figure 5:**
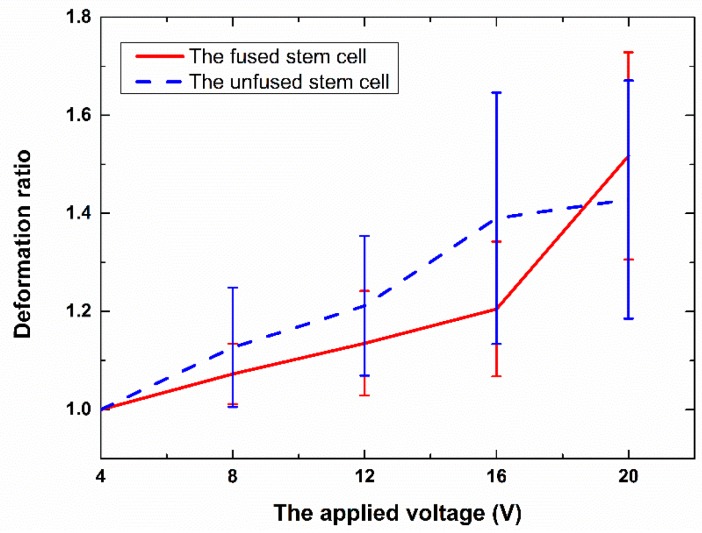
The deformation ratio vs. the applied voltage for the fused and unfused stem cells.

Figure 5 depicts the deformation ratio of the fused and unfused stem cells. For both types of cells, the deformation ratio increases as the applied voltage increases. However, the two cell types tested here were found to deform quite differently under identical ED conditions. The deformation ratios of the fused stem cell and unfused stem cell were, respectively, 1.205 ± 0.137 and 1.390 ± 0.256 at 16 V_p-p_, and 1.517 ± 0.211 and 1.428 ± 0.243 at 20 V_p-p_. No electrolysis was observed in the experiments. The fused stem cell was stiffer than the unfused stem cell at a relatively low voltage, and was less easily deformed by electrical stresses during ED. However, at 20 V, the fused stem cell was more easily deformed than the unfused stem cell. Above 20 V, the deformation of the fused stem cell was extremely large. This level of deformation may exceed the elastic limit of the fused stem cell, and some unwanted phenomena—such as electroporation on the cell membrane—occur. Because the mechanical properties of cells are largely determined by their cytoskeletons, this change may also arise from their different cytoskeletons.

For cell fusion, the largest radius of fused cells that we can obtain is $\sqrt[{3}]{2}R$, or $\sqrt{2}R$ after the fusion of two cells with radius *R*: (1) The volumes of the cells are assumed to be unchangeable, and the radius of the fused cell is $\sqrt[{3}]{2}R$; (2) the superficial areas of the cells are assumed to be unchangeable, and the radius of the fused cell is $\sqrt{2}R$. However, from our previous results, the radius of unfused stem cells is 7.55 ± 0.66 μm, but the radius of fused stem cells (8.88 ± 0.89 μm) is smaller than $\sqrt{2}R$ or $\sqrt[{3}]{2}R$. This result means that some part of the membrane of the cells was lost during the fusion process.

**Figure 6:**
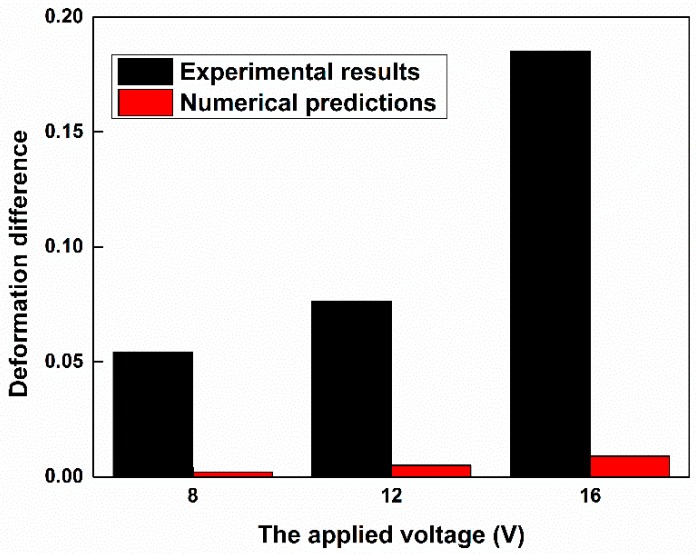
Deformation difference for the fused and unfused stem cells.

To compare with the experimental results, we assumed that the parameters of the cell membrane and cytoplasm were kept the same, and the same electric parameters of the fused and unfused cells were used in the simulation, except that the radius of the unfused stem cell was assumed to be 7.55 μm and the radius of the fused stem cell was assumed to be 8.88 μm. Note that a Young’s modulus of 1500 Pa was used to approximate the deformation ratio of the fused cell. Under the same conditions (16 V), the deformation ratio of the fused stem cell is approximately 1.212, which is smaller than that of the unfused stem cell (~1.221). The deformation difference is then 0.009, but from the experimental results, the deformation difference is ~0.185. Thus, compared to the experimental results, this deformation difference was due to the radius not being large enough to cause a significant difference to the deformation ratio of the fused and unfused cells (Figure 6). It could be concluded that compared to the unfused stem cell, not only was the radius of the fused stem cell changed, but also—and more importantly—the properties of the cell membrane were changed after fusion.

## 4. Conclusions

We present a method to analyze the deformability of fused cells. Electrical stresses, generated by a discrete co-planar microelectrode device with relatively low values of applied potential (4–20 V) have been used to electro-deform fused and unfused stem cells in suspension. The deformation ratios of the fused and unfused stem cells were, respectively, 1.205 ± 0.137 and 1.390 ± 0.256 at 16 V_p-p_, and 1.517 ± 0.211 and 1.428 ± 0.243 at 20 V_p-p_. Fused stem cells were stiffer than unfused stem cells at a relatively low voltage, and this trend was reversed at a relatively high voltage. It seems that the cytoskeletons of fused stem cells have been changed. The ED process is also modeled based on the Maxwell stress tensor and structural mechanics of cells. The simulation results showed that under the same conditions, the deformation ratio of fused stem cells is smaller than that of unfused stem cells, which is in qualitative agreement with the experimental observations. The numerical results also show that the significant difference in the deformation ratio of the fused and unfused stem cells is not due to their size difference; the change in deformability of the fused stem cells may be caused by the change in the cell membrane, and the properties of the cell membrane were changed after fusion.

## Figures and Tables

**Table 1:** Values of the constants and parameters used in the simulations.

Parameter	Value/Range	Reference
Medium conductivity ($\sigma_{\mathrm{out}}$)	0.001 S·m^−1^	Measured
Medium permittivity ($\varepsilon_{\mathrm{out}}$)	80	[23]
Cytoplasmic conductivity ($\sigma_{\mathrm{in}}$)	0.3 S·m^−1^	[23]
Cytoplasmic permittivity ($\varepsilon_{\mathrm{in}}$)	70	[18]
Cell membrane conductivity ($\sigma_{m}$)	5 × 10^−7^ S·m^−1^	[23]
Cell membrane permittivity ($\varepsilon_{m}$)	10	[18]
Cell membrane thickness ($d_{m}$)	5 nm	[23]
Young’s modulus	600 Pa	
Poisson’s ratio of cell (μ)	0.499	

## Abbreviations

The following abbreviations are used in this manuscript:

AC	Alternating current
ED	Electro-deformation
DEP	Dielectrophoresis
ER	Electrorotation
MPA	Micropipette aspiration
AFM	Atomic force microscopy
CCD	charge-coupled device
mESC	Mouse embryonic stem cell
H-DMEM	High-glucose dulbecco's modified eagle medium
EDTA	Ethylenediaminetetraacetic acid
FBS	Fetal bovine serum
PBS	Phosphate buffered saline
SOI	Silicon-on-insulator
